# Crystal structure of 2,4-di­amino-5-(4-hy­droxy-3-meth­oxy­phen­yl)-8,8-dimethyl-6-oxo-6,7,8,9-tetra­hydro-5*H*-chromeno[2,3-*b*]pyridine-3-carbo­nitrile–di­methyl­formamide–water (1/1/1)

**DOI:** 10.1107/S2056989024002615

**Published:** 2024-03-26

**Authors:** Nadia H. Metwally, Galal H. Elgemeie, El-shimaa S. M. Abd Al-latif, Peter G. Jones

**Affiliations:** a Chemistry Department, Faculty of Science, Cairo University, Giza, Egypt; b Chemistry Department, Faculty of Science, Helwan University, Cairo, Egypt; c Institut für Anorganische und Analytische Chemie, Technische Universität Braunschweig, Hagenring 30, D-38106 Braunschweig, Germany; Universität Greifswald, Germany

**Keywords:** crystal structure, chromeno­pyridine, solvate, secondary inter­actions

## Abstract

In the structure of the title compound, C_22_H_22_N_4_O_4_·C_3_H_7_NO·H_2_O, the entire tricyclic system is approximately planar except for the carbon atom bearing the two methyl groups; the meth­oxy­phenyl ring is approximately perpendicular to the tricycle. All seven potential hydrogen-bond donors take part in classical hydrogen bonds. The main mol­ecule and the DMF combine to form broad ribbons parallel to the *a* axis and roughly parallel to the *ab* plane; the water mol­ecules connect the residues in the third dimension.

## Abstract 2

The heterocyclic system of the title compound is approximately planar except for the carbon atom of the CMe_2_ group; the residues are connected by extensive classical hydrogen bonding.

## 1. Chemical context

Activated nitriles and α,β-unsaturated nitrile moieties are involved in a wide variety of natural plant products, drugs, colourants and agrochemicals (Fleming & Wang, 2003 ▸; Ahmed *et al.*, 2022 ▸); they also represent versatile starting materials for the synthesis of a wide variety of therapeutically important heterocycles (Zhang *et al.*, 2019 ▸; Metwally *et al.*, 2023 ▸). The generally accepted importance of these functions (Wang *et al.*, 2016 ▸; Hebishy *et al.*, 2023 ▸) is reflected in the investment of much effort to synthesize them (Zhang *et al.*, 2023 ▸; Elgemeie *et al.*, 1998*a* ▸,*b* ▸). Recently, we have reported several new methods for the synthesis of pharmaceutically relevant heterocycles utilizing activated nitriles and α,β-unsaturated nitriles as starting materials (*e.g.* Mohamed-Ezzat *et al.*, 2021 ▸). In this context, we and others have synthesized several condensed carbocyclic pyrans and carbocyclic pyridines using dimedone as the starting material (Hebishy *et al.*, 2022 ▸; Tu *et al.*, 2014 ▸). 
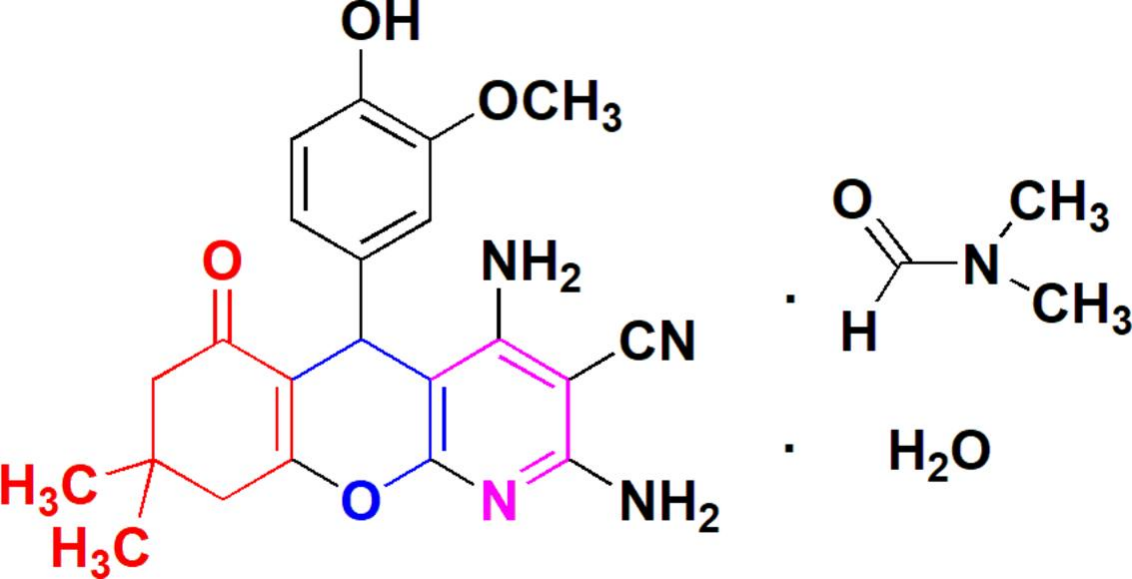


**Figure 1:**
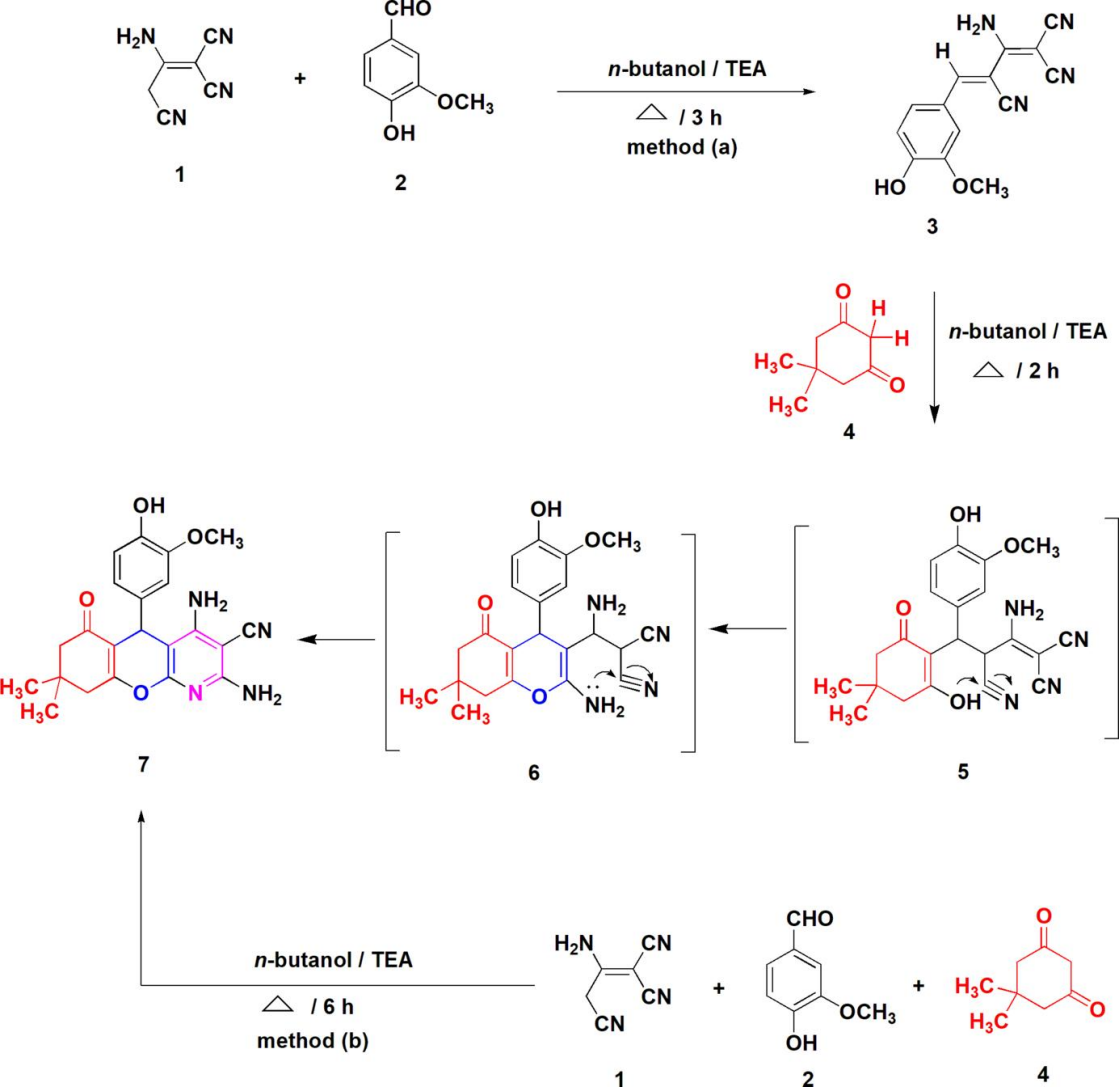
The reaction scheme for the synthesis of compound **7**.

The present investigation reports a new one-pot synthesis of condensed carbocyclic pyridines by the reaction of dimedone with enamino nitriles. It was found that 2-amino­prop-1-ene-1,1,3-tricarbo­nitrile (**1**) reacted with 4-hy­droxy-3-meth­oxy­benzaldehyde (**2**) and dimedone (**4**) in refluxing *n*-butanol containing catalytic amounts of tri­methyl­amine to give the corresponding condensed chromeno[2,3-*b*]pyridine-3-carbo­nitrile (**7**) (Fig. 1 ▸). The structure of **7** was confirmed on the basis of elemental analysis and spectroscopic studies (^1^H NMR, IR and MS). We suggest that the formation mechanism of **7** from **1**, **2** and **4** involves a condensation reaction that consists of an initial Michael addition of the methyl­ene group of the dimedone **4** to the double bond of inter­mediate **3** to give the next inter­mediate **5**, which then cyclizes to the condensed chromeno[2,3-*b*]pyridine-3-carbo­nitrile **7**. In order to establish the structure of the compound unambiguously, the crystal structure was determined and is presented here.

## 2. Structural commentary

**Figure 2:**
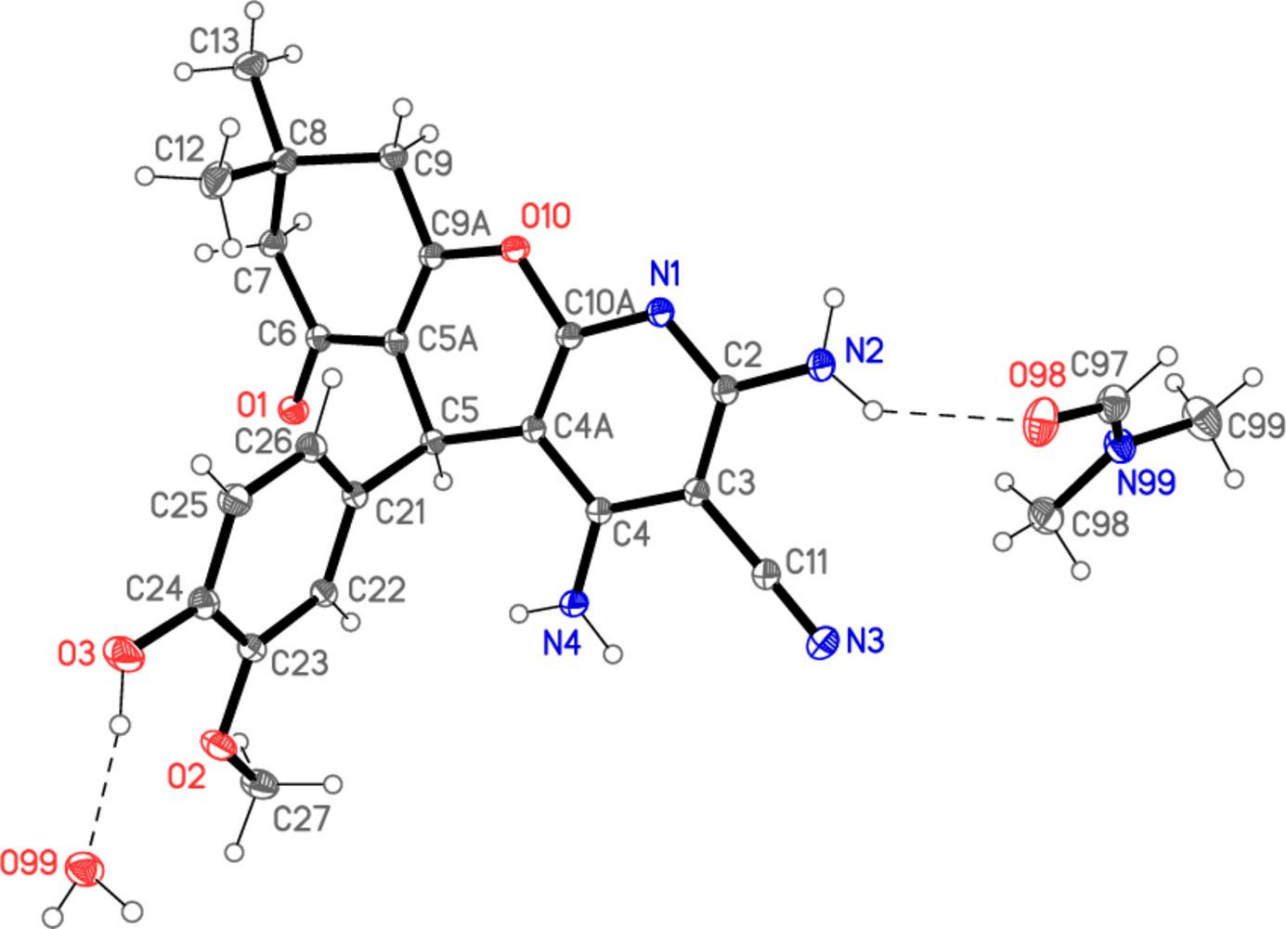
The structure of compound **7** (as its 1/1/1 adduct with DMF and water) in the crystal. Ellipsoids correspond to 50% probability levels. The dashed lines indicate hydrogen bonds.

**Figure 3:**
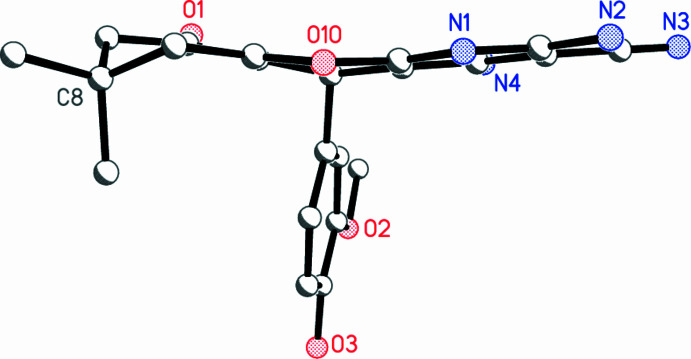
Side view of compound **7** (hydrogen atoms excluded).

The structure of the product **7**, which crystallized from DMF as a 1/1/1 adduct with DMF and water, is shown in Fig. 2 ▸. Mol­ecular dimensions, a selection of which are given in Table 1 ▸, may be regarded as normal (*e.g.* the double-bond length C5*A*=C9*A*). The pyridinic ring is planar, and its direct substituents also lie in the same plane (r.m.s. deviation of eleven atoms = 0.008 Å); the angle between this plane and that of the meth­oxy­phenyl ring is 77.86 (2)°. The atoms C5*A* and C9*A* lie 0.317 (1) and 0.249 (1) Å, respectively, out of the plane in the same direction. The central ring has the form of a flattened boat, with C5 and O10 lying 0.166 (1) and 0.101 (1) Å, respectively, out of the plane of the other four atoms (r.m.s. deviation = 0.015 Å). The third ring of the tricyclic system, formally related to cyclo­hexen-2-one, has the expected envelope form, in which the atom C8 lies 0.673 (1) Å out of the plane of the other five atoms (r.m.s. deviation 0.029 Å). Viewed from the side (Fig. 3 ▸), it can be seen that the entire tricyclic system is approximately planar (r.m.s. deviation 0.14 Å) except for C8.

## 3. Supra­molecular features

All seven of the potential hydrogen-bond donors do indeed take part in classical hydrogen bonds (Table 2 ▸), although the contact N4—H03⋯O98 is appreciably longer than the others, and O99—H07 is part of a three-centre system with N1 and the more distant O10 as acceptors. There is also one short linear contact involving a phenyl hydrogen, C22—H22⋯O98, which may be considered as a weak hydrogen bond; this is, however, not represented in the Figures for clarity reasons.

**Figure 4:**
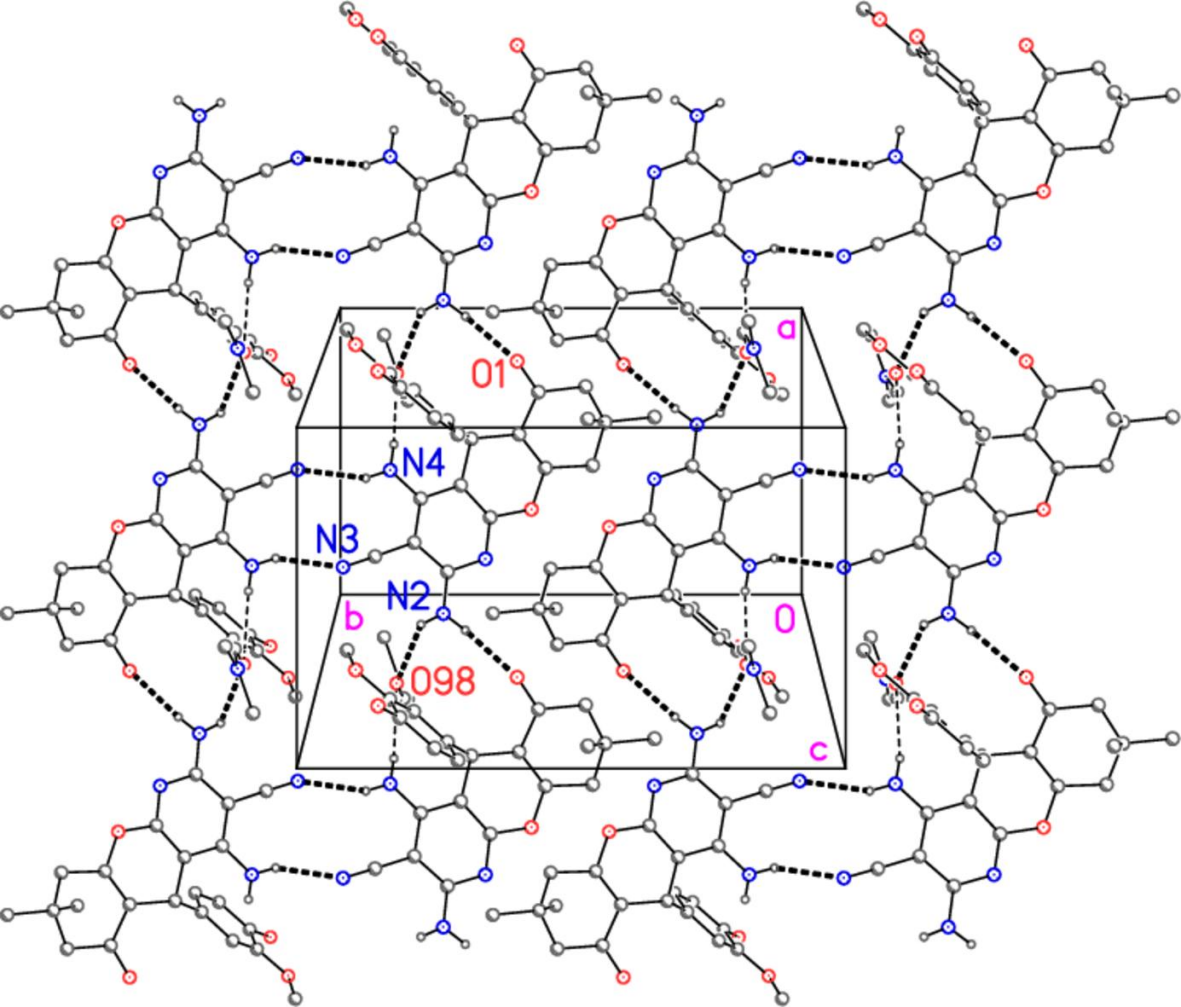
Packing diagram of compound **7** (including the DMF mol­ecules, which are seen edge-on), showing two broad ribbons running vertically. The meth­oxy­phenyl rings are reduced to the *ipso* atoms C21 for clarity. Hydrogen atoms not involved in hydrogen bonding are also omitted. View direction: perpendicular to the *ab* plane. Hydrogen bonds are shown as dashed lines (thin for the longer bonds N4—H03⋯O98, otherwise thick).

**Figure 5:**
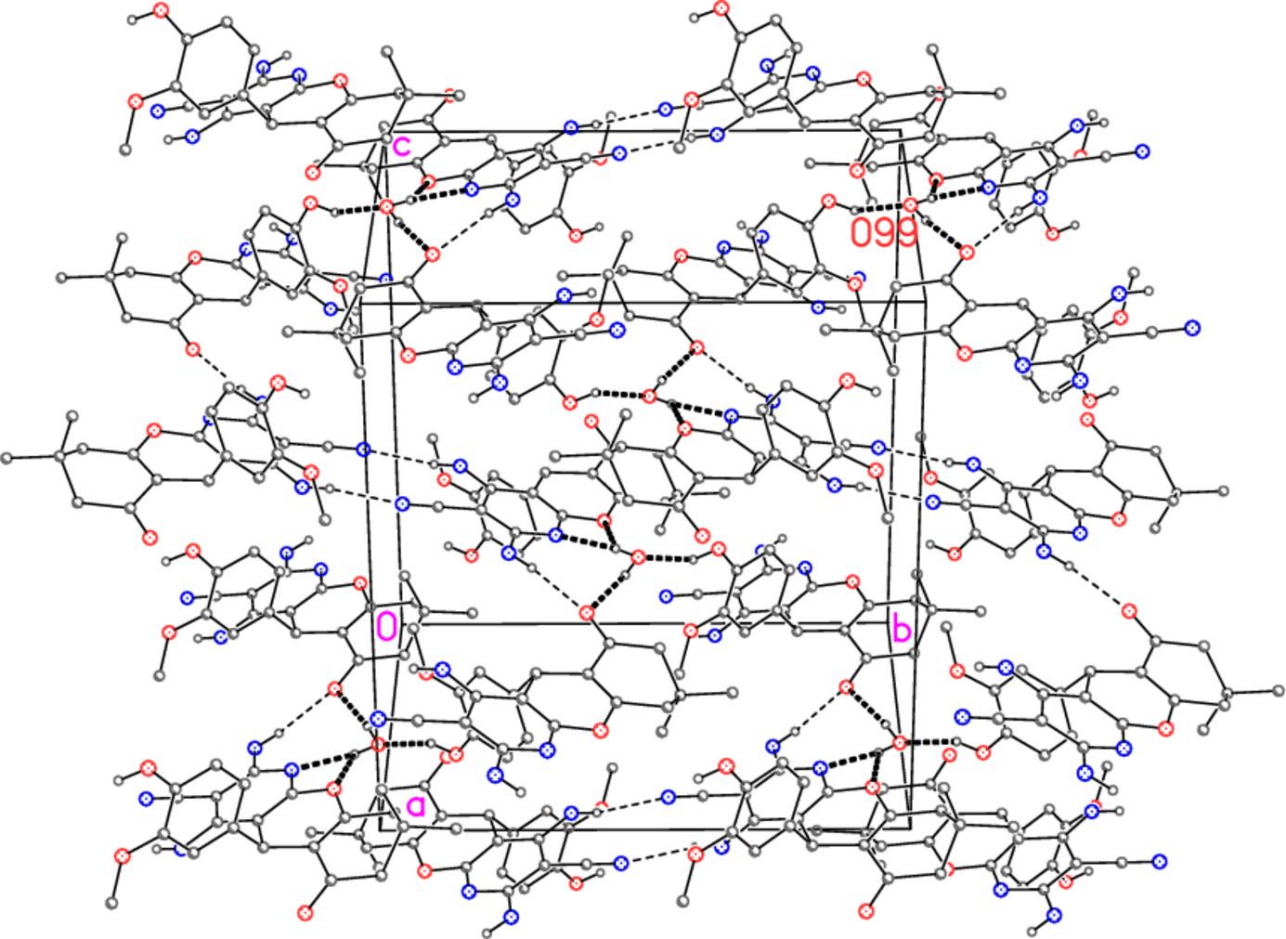
Packing diagram of compound **7**, with view direction approximately perpendicular to (101), showing the role of the water mol­ecules. Hydrogen bonds involving these mol­ecules are shown as thick dashed bonds, other hydrogen bonds as thin dashed bonds.

The mol­ecules of **7** and the DMF combine to form broad ribbons parallel to the *a* axis (Fig. 4 ▸), in which inversion-symmetric 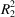 (12) rings, based on the hydrogen bond N4—H04⋯N3, are prominent. The DMF mol­ecules project above and below the planes of the ribbons. The water mol­ecules connect the residues in the third dimension (Fig. 5 ▸). They accept one hydrogen bond and act as donor for three hydrogen bonds (counting both branches of the three-centre system).

## 4. Database survey

The search employed the routine ConQuest (Bruno *et al.*, 2002 ▸), part of Version 2022.3.0 of the Cambridge Database (Groom *et al.*, 2016 ▸).

A search for the same tricyclic ring system gave only one hit, namely 8-(furan-2-yl)-12-(4-meth­oxy­phen­yl)-3,3,11-trimethyl-3,4,7,8,9,12-hexa­hydro-1*H*-chromeno[2,3-*b*]quinoline-1,10(2*H*)-dione (refcode EVANEW; Han *et al.*, 2015 ▸). This, however, has a further cyclo­hexa­none-type ring fused to the pyridinic ring. In common with compound **7**, it bears two methyl groups at the atom corresponding to our C8, a keto function at C6 and an aromatic substituent (*p*-meth­oxy­phen­yl) at C5. The inter­planar angle involving this ring is given as 83.7 (7)°.

A search for solvates with precisely one DMF and one water mol­ecule (under the stringent conditions only organic, no disorder, no ionic compounds, no metals, all solvent H present) gave only 32 hits. We did not check for the plausibility of the water H atoms. The structures represented a broad distribution of organic compounds, *e.g.* during systematic studies of solvates of crown ethers [17,23-di­bromo-18,22-di­nitro-2,5,8,11,14-penta­oxa-26-aza­tetra­cyclo-(13.9.3.019,27.021,25)hepta­cosa-1(24),15,17,19 (27),21 (25),22-hexa­ene-20(26*H*)-one, refcode AMARAH; Huszthy *et al.*, 2003 ▸] or steroids [bis­(17β-hy­droxy-17α-methyl­androstano[3,2-*c*]pyrazole, AVEQUO; Karpinska *et al.*, 2011 ▸]. Heterocyclic systems with groups likely to hydrogen bond were also well represented, *e.g.* 2′-amino-6′-ethyl-2,5′-dioxo-1,2,5′,6′-tetra­hydro­spiro­[indole-3,4′-pyrano[3,2-*c*]quinoline]-3′-carbo­nitrile (MESVAL; Upadhyay *et al.*, 2023 ▸). Our own studies have shown that DMF is often a useful solvent for crystallization of heterocyclic compounds; as a hydrogen-bond acceptor, it has formed solvates with *N*-[2-amino-5-cyano-4-(methyl­sulfan­yl)-6-oxopyrimidin-1(6*H*)-yl]-4-bromo­benzene­sulfonamide (WUSMUU; Elgemeie *et al.*, 2015 ▸) and *N*-[6-amino-5-(1,3-benzo­thia­zol-2-yl)-3-cyano-4-(methyl­sulfan­yl)-2-oxopyridin-1(2*H*)-yl]-4-methyl­benzene-1-sulfonamide (ZELBUQ; Azzam *et al.*, 2017 ▸).

## 5. Synthesis and crystallization

**Method A**

A mixture of 2-amino­prop-1-ene-1,1,3-tricarbo­nitrile **1** (1.32 g, 0.01 mmol), 4-hy­droxy-3-meth­oxy­benzaldehyde **2** (1.52 g, 0.01 mmol) and a few drops of tri­ethyl­amine in *n*-butanol (50 mL) was refluxed for 3 h. Then 5,5-di­methyl­cyclo­hexane-1,3-dione **4** (1.4 g, 0.01 mmol) was added and the mixture was refluxed for another 2 h. After cooling, the precipitate was collected by filtration and recrystallized from DMF. Yield 2.84 g (70%).

**Method B**

A mixture of 2-amino­prop-1-ene-1,1,3-tricarbo­nitrile **1** (1.32 g, 0.01 mmol), 4-hy­droxy-3-meth­oxy­benzaldehyde **2** (1.52 g, 0.01 m mol), 5,5-di­methyl­cyclo­hexane-1,3-dione **4** (1.4 g, 0.01 mmol) and few drops of tri­ethyl­amine in *n*-butanol (5 ml) was refluxed for 6 h. After cooling, the precipitate was collected by filtration and recrystallized from DMF. Yield 3.04 g (75%).

Orange crystals, yield 75%, m.p. 516–518 K. IR (KBr): ν_max_ = 3448 (OH), 3351(NH_2_), 2204 (CN), 1662 (C=O) cm^−1^; ^1^H NMR (400 MHz, DMSO-*d*_6_): δ = 1.00 (*s*, 3H,CH_3_), 1.05 (*s*, 3H, CH_3_), 2.43–2.52 (*m*, 4H, 2 CH_2_), 3.66 (*s*, 3H,OCH_3_), 4.75 (*s*, 1H, pyran-H), 6.38–6.42 (*m*, 5H, Ar-1H and 2 NH_2_), 6.96 (*s*, 1H, Ar), 7.92 (*s*, 1H, Ar), 8.71 (*s*, 1H, OH) ppm. ^13^C NMR (100 MHz, DMSO-*d*_6_): δ = 196.19 (C=O), 164.06 (O—C—N), 162.84 (C—O), 159.72 (C—NH_2_), 157.22 (N=C—NH_2_), 147.36 (C—OCH_3_), 145.52 (C—OH), 135.72 (Ar—C), 120.37, 116.67 (Ar—CH), 115.07 (CN), 92.51 (C—CO), 72.13 (pyridine-C), 56.21(C—CN), 50.68 (OCH_3_), 33.29 (CH_2_), 32.56 (CH_2_), 29.32 (CH_3_), 26.83 (CH_3_) ppm. MS (70 eV, Fab mass, %): *m*/*z* = 406 (11%), 372 (9), 282 (100), 226 (33), 170 (11), 66 (9). Analysis calculated for C_22_H_22_N_4_O_4_ (406.16): C 65.01, H 5.46, N 13.78. Found: C 65.0, H 5.5, N 13.7%.

## 6. Refinement

Crystal data, data collection and structure refinement details are summarized in Table 3 ▸. Hydrogen atoms bonded to nitro­gen or oxygen were refined freely. The methyl groups were included as an idealized rigid group allowed to rotate but not tip (command AFIX 137), with C—H = 0.99 Å and H—C—H = 109.5°. Other hydrogen atoms were included using a riding model starting from calculated positions (C—H_methyl­ene_ = 0.99, C—H_methine_ = 1.00, C—H_arom_ = 0.95 Å). The *U*(H) values were fixed at 1.5 × *U*_eq_ of the parent carbon atoms for the methyl groups and 1.2 × *U*_eq_ for other hydrogens. Three reflections, with intensities clearly in error, were omitted. The largest peaks of residual electron density (max. 0.67 e Å^−3^) lie in the middle of bonds and thus do not give cause for concern.

## Supplementary Material

Crystal structure: contains datablock(s) I, global. DOI: 10.1107/S2056989024002615/yz2052sup1.cif

Structure factors: contains datablock(s) I. DOI: 10.1107/S2056989024002615/yz2052Isup2.hkl

Supporting information file. DOI: 10.1107/S2056989024002615/yz2052Isup3.cml

CCDC reference: 2341559

Additional supporting information:  crystallographic information; 3D view; checkCIF report

## Figures and Tables

**Table 1:** Selected geometric parameters (Å, °)

N1—C10*A*	1.3324 (7)	C9*A*—O10	1.3617 (7)
N1—C2	1.3399 (7)	O10—C10*A*	1.3783 (7)
C5*A*—C9*A*	1.3484 (8)		
			
C10*A*—N1—C2	117.21 (5)	C4*A*—C10*A*—O10	122.17 (5)
			
C10*A*—C4*A*—C5—C5*A*	11.45 (7)	C5—C5*A*—C9*A*—O10	5.67 (9)
C4*A*—C5—C5*A*—C9*A*	−14.61 (7)	C6—C5*A*—C9*A*—C9	5.77 (9)
C9*A*—C5*A*—C6—C7	−8.71 (8)	C8—C9—C9*A*—C5*A*	26.39 (9)
C5*A*—C6—C7—C8	−21.02 (8)	C5*A*—C9*A*—O10—C10*A*	8.25 (9)
C6—C7—C8—C9	50.42 (7)	C5—C4*A*—C10*A*—O10	0.48 (9)
C7—C8—C9—C9*A*	−51.84 (7)	C9*A*—O10—C10*A*—C4*A*	−11.26 (9)

**Table 2:** Hydrogen-bond geometry (Å, °)

*D*—H⋯*A*	*D*—H	H⋯*A*	*D*⋯*A*	*D*—H⋯*A*
N2—H01⋯O1^i^	0.886 (13)	2.268 (13)	3.1492 (7)	173.6 (11)
N2—H02⋯O98	0.885 (13)	2.170 (13)	2.9167 (8)	141.8 (11)
N4—H03⋯O98^ii^	0.836 (13)	2.590 (12)	3.2891 (8)	142.0 (11)
N4—H04⋯N3^iii^	0.863 (13)	2.237 (13)	3.0413 (8)	154.9 (12)
O3—H05⋯O99	0.879 (14)	1.816 (14)	2.6399 (7)	155.2 (12)
O99—H06⋯O1^iv^	0.856 (14)	1.944 (14)	2.7944 (7)	172.0 (13)
O99—H07⋯N1^v^	0.877 (14)	2.012 (14)	2.8699 (7)	165.5 (13)
O99—H07⋯O10^v^	0.877 (14)	2.519 (14)	3.2180 (7)	137.2 (11)
C22—H22⋯O98^ii^	0.95	2.39	3.3210 (8)	167

Symmetry codes: (i) 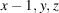 ; (ii) 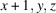 ; (iii) 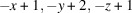 ; (iv) 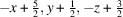 ; (v) 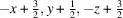 .

**Table 3:** Experimental details

Crystal data	
Chemical formula	C_22_H_22_N_4_O_4_·C_3_H_7_NO·H_2_O
*M*_r_	497.55
Crystal system, space group	Monoclinic, *P*2_1_/*n*
Temperature (K)	100
*a*, *b*, *c* (Å)	9.9055 (2), 15.9600 (3), 16.4794 (4)
β (°)	106.344 (2)
*V* (Å^3^)	2499.98 (10)
*Z*	4
Radiation type	Mo *K*α
μ (mm^−1^)	0.10
Crystal size (mm)	0.2 × 0.2 × 0.1

Data collection	
Diffractometer	XtaLAB Synergy
Absorption correction	Multi-scan (*CrysAlis PRO*; Rigaku OD, 2022 ▸)
*T*_min_, *T*_max_	0.735, 1.000
No. of measured, independent and observed [*I* > 2σ(*I*)] reflections	210685, 13416, 11108
*R*_int_	0.056
θ values (°)	θ_max_ = 37.8, θ_min_ = 2.2
(sin θ/λ)_max_ (Å^−1^)	0.862

Refinement	
*R*[*F* ^2^ > 2σ(*F* ^2^)], *wR*(*F* ^2^), *S*	0.038, 0.114, 1.04
No. of reflections	13416
No. of parameters	358
H-atom treatment	H atoms treated by a mixture of independent and constrained refinement
Δρ_max_, Δρ_min_ (e Å^−3^)	0.67, −0.22

Computer programs: *CrysAlis PRO* (Rigaku OD, 2022 ▸), *SHELXT* (Sheldrick, 2015*a* ▸), *SHELXL2019/3* (Sheldrick, 2015*b* ▸) and *XP* (Bruker, 1998 ▸).

## supplementary crystallographic information

### Crystal data

**Table:**

C_22_H_22_N_4_O_4_·C_3_H_7_NO·H_2_O	*F*(000) = 1056
*M**_r_* = 497.55	*D*_x_ = 1.322 Mg m^−^^3^
Monoclinic, *P*2_1_/*n*	Mo *K*α radiation, λ = 0.71073 Å
*a* = 9.9055 (2) Å	Cell parameters from 76991 reflections
*b* = 15.9600 (3) Å	θ = 2.2–39.6°
*c* = 16.4794 (4) Å	µ = 0.10 mm^−^^1^
β = 106.344 (2)°	*T* = 100 K
*V* = 2499.98 (10) Å^3^	Tablet, colourless
*Z* = 4	0.2 × 0.2 × 0.1 mm

### Data collection

**Table:**

XtaLAB Synergy diffractometer	13416 independent reflections
Radiation source: micro-focus sealed X-ray tube, PhotonJet (Mo) X-ray Source	11108 reflections with *I* > 2σ(*I*)
Mirror monochromator	*R*_int_ = 0.056
Detector resolution: 10.0000 pixels mm^-1^	θ_max_ = 37.8°, θ_min_ = 2.2°
ω scans	*h* = −17→17
Absorption correction: multi-scan (CrysAlisPro; Rigaku OD, 2022)	*k* = −27→27
*T*_min_ = 0.735, *T*_max_ = 1.000	*l* = −28→28
210685 measured reflections	

### Refinement

**Table:**

Refinement on *F*^2^	Primary atom site location: dual
Least-squares matrix: full	Hydrogen site location: mixed
*R*[*F*^2^ > 2σ(*F*^2^)] = 0.038	H atoms treated by a mixture of independent and constrained refinement
*wR*(*F*^2^) = 0.114	*w* = 1/[σ^2^(*F*_o_^2^) + (0.0626*P*)^2^ + 0.4082*P*] where *P* = (*F*_o_^2^ + 2*F*_c_^2^)/3
*S* = 1.04	(Δ/σ)_max_ = 0.001
13416 reflections	Δρ_max_ = 0.67 e Å^−^^3^
358 parameters	Δρ_min_ = −0.22 e Å^−^^3^
0 restraints	

### Fractional atomic coordinates and isotropic or equivalent isotropic displacement parameters (Å^2^)

**Table:**

	*x*	*y*	*z*	*U*_iso_*/*U*_eq_	
N1	0.41040 (5)	0.66845 (3)	0.58286 (3)	0.01573 (8)	
C2	0.35681 (6)	0.74408 (3)	0.55675 (4)	0.01397 (8)	
N2	0.21622 (5)	0.75300 (4)	0.54244 (4)	0.01919 (9)	
H01	0.1683 (13)	0.7091 (8)	0.5522 (8)	0.029 (3)*	
H02	0.1692 (13)	0.7983 (8)	0.5197 (8)	0.032 (3)*	
C3	0.44457 (6)	0.81052 (3)	0.54550 (3)	0.01332 (8)	
C4	0.59114 (6)	0.79689 (3)	0.55989 (3)	0.01270 (8)	
N4	0.67486 (5)	0.85813 (3)	0.54512 (4)	0.01630 (9)	
H03	0.7612 (13)	0.8503 (8)	0.5540 (8)	0.030 (3)*	
H04	0.6425 (14)	0.9076 (8)	0.5296 (8)	0.032 (3)*	
C4A	0.64624 (5)	0.71688 (3)	0.58823 (3)	0.01254 (8)	
C5	0.80070 (5)	0.69551 (3)	0.60532 (3)	0.01224 (8)	
H5	0.832899	0.714157	0.555889	0.015*	
C5A	0.81825 (6)	0.60167 (3)	0.61409 (3)	0.01306 (8)	
C6	0.94969 (6)	0.56393 (3)	0.60992 (4)	0.01383 (8)	
O1	1.03852 (5)	0.60592 (3)	0.58804 (3)	0.01802 (8)	
C7	0.97538 (6)	0.47181 (4)	0.63057 (4)	0.01648 (9)	
H7A	1.075931	0.463992	0.661303	0.020*	
H7B	0.956598	0.440073	0.576928	0.020*	
C8	0.88588 (6)	0.43440 (4)	0.68387 (4)	0.01705 (9)	
C9	0.73204 (6)	0.45817 (4)	0.64135 (4)	0.01770 (10)	
H9A	0.698047	0.427626	0.587129	0.021*	
H9B	0.673250	0.441184	0.678188	0.021*	
C9A	0.71700 (6)	0.55019 (3)	0.62522 (4)	0.01452 (9)	
O10	0.58553 (5)	0.57747 (3)	0.62246 (3)	0.01756 (8)	
C10A	0.54867 (6)	0.65861 (3)	0.59701 (4)	0.01403 (9)	
C11	0.38620 (6)	0.89004 (4)	0.51709 (4)	0.01655 (9)	
N3	0.34038 (7)	0.95535 (4)	0.49419 (5)	0.02514 (12)	
C12	0.93212 (8)	0.46813 (5)	0.77471 (4)	0.02391 (12)	
H12A	1.026948	0.447741	0.803183	0.036*	
H12B	0.866481	0.448682	0.805418	0.036*	
H12C	0.932458	0.529524	0.773538	0.036*	
C13	0.90238 (8)	0.33896 (4)	0.68645 (6)	0.02501 (13)	
H13A	0.867471	0.316360	0.629044	0.038*	
H13B	0.848265	0.314956	0.722263	0.038*	
H13C	1.001952	0.324471	0.709689	0.038*	
C21	0.89109 (5)	0.73909 (3)	0.68486 (3)	0.01264 (8)	
C22	0.98270 (6)	0.80413 (3)	0.67880 (3)	0.01367 (8)	
H22	0.991495	0.819834	0.624914	0.016*	
C23	1.06120 (6)	0.84614 (4)	0.75094 (4)	0.01489 (9)	
O2	1.15118 (5)	0.91117 (3)	0.75031 (3)	0.01993 (9)	
C24	1.05186 (6)	0.82200 (4)	0.83128 (4)	0.01673 (9)	
O3	1.12872 (6)	0.85824 (4)	0.90394 (3)	0.02564 (11)	
H05	1.1648 (14)	0.9072 (8)	0.8971 (8)	0.034 (3)*	
C25	0.96244 (7)	0.75654 (4)	0.83672 (4)	0.01757 (10)	
H25	0.955884	0.739310	0.890663	0.021*	
C26	0.88214 (6)	0.71565 (4)	0.76446 (4)	0.01552 (9)	
H26	0.820851	0.671449	0.769635	0.019*	
C27	1.16872 (8)	0.93355 (4)	0.67030 (4)	0.02151 (11)	
H27A	1.077008	0.947598	0.631269	0.032*	
H27B	1.209836	0.886314	0.647569	0.032*	
H27C	1.231375	0.982140	0.676969	0.032*	
C97	−0.12972 (7)	0.83937 (4)	0.40895 (4)	0.02086 (11)	
H97	−0.220125	0.819639	0.409112	0.025*	
C98	0.02521 (8)	0.88437 (5)	0.32596 (5)	0.02539 (13)	
H98A	0.082462	0.904757	0.380990	0.038*	
H98B	0.008664	0.930221	0.284779	0.038*	
H98C	0.074841	0.838697	0.306776	0.038*	
O98	−0.04221 (6)	0.84926 (4)	0.47838 (3)	0.02704 (11)	
N99	−0.10846 (6)	0.85392 (4)	0.33387 (3)	0.01883 (9)	
C99	−0.21966 (8)	0.83914 (6)	0.25620 (5)	0.02841 (14)	
H99A	−0.193276	0.792258	0.225408	0.043*	
H99B	−0.233445	0.889604	0.220932	0.043*	
H99C	−0.307232	0.825602	0.269909	0.043*	
O99	1.23412 (5)	1.01108 (3)	0.92936 (3)	0.02037 (9)	
H06	1.3078 (14)	1.0356 (8)	0.9237 (8)	0.033 (3)*	
H07	1.1765 (15)	1.0538 (9)	0.9237 (9)	0.039 (3)*	

### Atomic displacement parameters (Å^2^)

**Table:**

	*U*^11^	*U*^22^	*U*^33^	*U*^12^	*U*^13^	*U*^23^
N1	0.01093 (17)	0.01421 (18)	0.0225 (2)	0.00127 (14)	0.00540 (16)	0.00266 (15)
C2	0.01156 (19)	0.0146 (2)	0.0158 (2)	0.00106 (15)	0.00391 (16)	0.00067 (16)
N2	0.01142 (19)	0.0177 (2)	0.0285 (3)	0.00216 (16)	0.00582 (17)	0.00442 (18)
C3	0.01208 (19)	0.01273 (19)	0.0148 (2)	0.00120 (15)	0.00318 (15)	0.00130 (15)
C4	0.01184 (19)	0.01280 (19)	0.01307 (19)	0.00009 (15)	0.00285 (15)	0.00058 (15)
N4	0.01292 (18)	0.01354 (18)	0.0221 (2)	−0.00014 (15)	0.00446 (16)	0.00403 (16)
C4A	0.01060 (18)	0.01223 (18)	0.01442 (19)	0.00021 (14)	0.00292 (15)	0.00092 (15)
C5	0.01039 (18)	0.01210 (18)	0.01403 (19)	−0.00016 (14)	0.00309 (15)	0.00053 (15)
C5A	0.01084 (18)	0.01237 (18)	0.0157 (2)	0.00037 (15)	0.00333 (15)	0.00069 (15)
C6	0.01133 (19)	0.0143 (2)	0.0155 (2)	0.00046 (15)	0.00311 (16)	0.00048 (16)
O1	0.01310 (17)	0.01693 (18)	0.0254 (2)	−0.00010 (14)	0.00763 (15)	0.00243 (15)
C7	0.0139 (2)	0.0144 (2)	0.0213 (2)	0.00266 (16)	0.00530 (18)	0.00277 (17)
C8	0.0144 (2)	0.0142 (2)	0.0224 (2)	0.00181 (17)	0.00503 (18)	0.00436 (18)
C9	0.0133 (2)	0.0129 (2)	0.0266 (3)	0.00028 (16)	0.00501 (19)	0.00304 (18)
C9A	0.01132 (19)	0.01308 (19)	0.0190 (2)	0.00082 (15)	0.00398 (16)	0.00187 (16)
O10	0.01171 (16)	0.01288 (16)	0.0291 (2)	0.00140 (13)	0.00737 (15)	0.00510 (15)
C10A	0.01180 (19)	0.01265 (19)	0.0178 (2)	0.00074 (15)	0.00446 (16)	0.00174 (16)
C11	0.0130 (2)	0.0159 (2)	0.0198 (2)	0.00068 (16)	0.00297 (17)	0.00216 (17)
N3	0.0186 (2)	0.0177 (2)	0.0366 (3)	0.00268 (18)	0.0038 (2)	0.0075 (2)
C12	0.0241 (3)	0.0267 (3)	0.0200 (3)	0.0026 (2)	0.0046 (2)	0.0068 (2)
C13	0.0210 (3)	0.0150 (2)	0.0396 (4)	0.0037 (2)	0.0094 (3)	0.0076 (2)
C21	0.01090 (18)	0.01291 (19)	0.01364 (19)	−0.00027 (15)	0.00269 (15)	0.00041 (15)
C22	0.01250 (19)	0.0141 (2)	0.01379 (19)	−0.00121 (15)	0.00278 (15)	0.00045 (15)
C23	0.0142 (2)	0.0144 (2)	0.0151 (2)	−0.00214 (16)	0.00256 (16)	0.00023 (16)
O2	0.0216 (2)	0.01943 (19)	0.01761 (18)	−0.00873 (16)	0.00363 (15)	−0.00127 (15)
C24	0.0180 (2)	0.0169 (2)	0.0136 (2)	−0.00189 (18)	0.00167 (17)	−0.00018 (17)
O3	0.0343 (3)	0.0243 (2)	0.01417 (18)	−0.0115 (2)	0.00005 (18)	−0.00124 (16)
C25	0.0201 (2)	0.0185 (2)	0.0137 (2)	−0.00243 (19)	0.00417 (18)	0.00106 (17)
C26	0.0154 (2)	0.0158 (2)	0.0153 (2)	−0.00179 (17)	0.00414 (17)	0.00110 (16)
C27	0.0242 (3)	0.0197 (2)	0.0215 (3)	−0.0072 (2)	0.0078 (2)	−0.0002 (2)
C97	0.0195 (2)	0.0257 (3)	0.0172 (2)	0.0028 (2)	0.0049 (2)	0.0038 (2)
C98	0.0200 (3)	0.0277 (3)	0.0303 (3)	−0.0008 (2)	0.0100 (2)	0.0045 (2)
O98	0.0258 (2)	0.0361 (3)	0.0163 (2)	0.0069 (2)	0.00111 (17)	0.00401 (18)
N99	0.0165 (2)	0.0236 (2)	0.0159 (2)	−0.00074 (17)	0.00382 (16)	0.00215 (17)
C99	0.0252 (3)	0.0405 (4)	0.0169 (3)	−0.0059 (3)	0.0015 (2)	−0.0013 (2)
O99	0.01655 (19)	0.01737 (19)	0.0278 (2)	−0.00084 (15)	0.00723 (17)	−0.00182 (16)

### Geometric parameters (Å, º)

**Table:**

N1—C10A	1.3324 (7)	C12—H12B	0.9800
N1—C2	1.3399 (7)	C12—H12C	0.9800
C2—N2	1.3529 (8)	C13—H13A	0.9800
C2—C3	1.4160 (8)	C13—H13B	0.9800
N2—H01	0.886 (13)	C13—H13C	0.9800
N2—H02	0.885 (13)	C21—C26	1.3907 (8)
C3—C11	1.4183 (8)	C21—C22	1.4007 (8)
C3—C4	1.4204 (8)	C22—C23	1.3954 (8)
C4—N4	1.3476 (7)	C22—H22	0.9500
C4—C4A	1.4154 (7)	C23—O2	1.3700 (7)
N4—H03	0.836 (13)	C23—C24	1.4068 (8)
N4—H04	0.863 (13)	O2—C27	1.4230 (8)
C4A—C10A	1.3779 (8)	C24—O3	1.3545 (8)
C4A—C5	1.5146 (7)	C24—C25	1.3888 (9)
C5—C5A	1.5101 (7)	O3—H05	0.879 (14)
C5—C21	1.5308 (8)	C25—C26	1.3941 (8)
C5—H5	1.0000	C25—H25	0.9500
C5A—C9A	1.3484 (8)	C26—H26	0.9500
C5A—C6	1.4535 (8)	C27—H27A	0.9800
C6—O1	1.2377 (7)	C27—H27B	0.9800
C6—C7	1.5145 (8)	C27—H27C	0.9800
C7—C8	1.5337 (9)	C97—O98	1.2359 (8)
C7—H7A	0.9900	C97—N99	1.3326 (9)
C7—H7B	0.9900	C97—H97	0.9500
C8—C13	1.5313 (9)	C98—N99	1.4503 (9)
C8—C12	1.5345 (10)	C98—H98A	0.9800
C8—C9	1.5349 (8)	C98—H98B	0.9800
C9—C9A	1.4925 (8)	C98—H98C	0.9800
C9—H9A	0.9900	N99—C99	1.4538 (9)
C9—H9B	0.9900	C99—H99A	0.9800
C9A—O10	1.3617 (7)	C99—H99B	0.9800
O10—C10A	1.3783 (7)	C99—H99C	0.9800
C11—N3	1.1570 (8)	O99—H06	0.856 (14)
C12—H12A	0.9800	O99—H07	0.877 (14)
			
C10A—N1—C2	117.21 (5)	C8—C12—H12A	109.5
N1—C2—N2	116.49 (5)	C8—C12—H12B	109.5
N1—C2—C3	120.95 (5)	H12A—C12—H12B	109.5
N2—C2—C3	122.56 (5)	C8—C12—H12C	109.5
C2—N2—H01	117.6 (8)	H12A—C12—H12C	109.5
C2—N2—H02	123.6 (8)	H12B—C12—H12C	109.5
H01—N2—H02	118.6 (11)	C8—C13—H13A	109.5
C2—C3—C11	120.33 (5)	C8—C13—H13B	109.5
C2—C3—C4	119.98 (5)	H13A—C13—H13B	109.5
C11—C3—C4	119.65 (5)	C8—C13—H13C	109.5
N4—C4—C4A	120.95 (5)	H13A—C13—H13C	109.5
N4—C4—C3	120.71 (5)	H13B—C13—H13C	109.5
C4A—C4—C3	118.32 (5)	C26—C21—C22	118.77 (5)
C4—N4—H03	120.8 (9)	C26—C21—C5	120.55 (5)
C4—N4—H04	121.4 (9)	C22—C21—C5	120.68 (5)
H03—N4—H04	117.7 (12)	C23—C22—C21	120.81 (5)
C10A—C4A—C4	115.27 (5)	C23—C22—H22	119.6
C10A—C4A—C5	122.02 (5)	C21—C22—H22	119.6
C4—C4A—C5	122.69 (5)	O2—C23—C22	124.42 (5)
C5A—C5—C4A	108.92 (4)	O2—C23—C24	115.46 (5)
C5A—C5—C21	110.17 (4)	C22—C23—C24	120.12 (5)
C4A—C5—C21	111.95 (4)	C23—O2—C27	116.52 (5)
C5A—C5—H5	108.6	O3—C24—C25	118.31 (5)
C4A—C5—H5	108.6	O3—C24—C23	123.06 (6)
C21—C5—H5	108.6	C25—C24—C23	118.61 (5)
C9A—C5A—C6	117.67 (5)	C24—O3—H05	114.5 (9)
C9A—C5A—C5	123.19 (5)	C24—C25—C26	121.20 (5)
C6—C5A—C5	119.14 (5)	C24—C25—H25	119.4
O1—C6—C5A	120.61 (5)	C26—C25—H25	119.4
O1—C6—C7	120.18 (5)	C21—C26—C25	120.48 (5)
C5A—C6—C7	119.19 (5)	C21—C26—H26	119.8
C6—C7—C8	114.77 (5)	C25—C26—H26	119.8
C6—C7—H7A	108.6	O2—C27—H27A	109.5
C8—C7—H7A	108.6	O2—C27—H27B	109.5
C6—C7—H7B	108.6	H27A—C27—H27B	109.5
C8—C7—H7B	108.6	O2—C27—H27C	109.5
H7A—C7—H7B	107.6	H27A—C27—H27C	109.5
C13—C8—C7	109.19 (5)	H27B—C27—H27C	109.5
C13—C8—C12	108.77 (6)	O98—C97—N99	125.77 (7)
C7—C8—C12	111.11 (5)	O98—C97—H97	117.1
C13—C8—C9	110.06 (5)	N99—C97—H97	117.1
C7—C8—C9	107.58 (5)	N99—C98—H98A	109.5
C12—C8—C9	110.12 (5)	N99—C98—H98B	109.5
C9A—C9—C8	111.17 (5)	H98A—C98—H98B	109.5
C9A—C9—H9A	109.4	N99—C98—H98C	109.5
C8—C9—H9A	109.4	H98A—C98—H98C	109.5
C9A—C9—H9B	109.4	H98B—C98—H98C	109.5
C8—C9—H9B	109.4	C97—N99—C98	121.91 (6)
H9A—C9—H9B	108.0	C97—N99—C99	120.70 (6)
C5A—C9A—O10	122.79 (5)	C98—N99—C99	117.39 (6)
C5A—C9A—C9	125.66 (5)	N99—C99—H99A	109.5
O10—C9A—C9	111.55 (5)	N99—C99—H99B	109.5
C9A—O10—C10A	118.66 (5)	H99A—C99—H99B	109.5
N1—C10A—C4A	128.24 (5)	N99—C99—H99C	109.5
N1—C10A—O10	109.59 (5)	H99A—C99—H99C	109.5
C4A—C10A—O10	122.17 (5)	H99B—C99—H99C	109.5
N3—C11—C3	179.08 (6)	H06—O99—H07	100.5 (12)
			
C10A—N1—C2—N2	179.82 (6)	C6—C5A—C9A—C9	5.77 (9)
C10A—N1—C2—C3	−0.47 (9)	C5—C5A—C9A—C9	−174.65 (6)
N1—C2—C3—C11	179.43 (6)	C8—C9—C9A—C5A	26.39 (9)
N2—C2—C3—C11	−0.88 (9)	C8—C9—C9A—O10	−153.90 (5)
N1—C2—C3—C4	1.58 (8)	C5A—C9A—O10—C10A	8.25 (9)
N2—C2—C3—C4	−178.73 (6)	C9—C9A—O10—C10A	−171.47 (5)
C2—C3—C4—N4	176.42 (5)	C2—N1—C10A—C4A	−0.02 (9)
C11—C3—C4—N4	−1.44 (8)	C2—N1—C10A—O10	−179.40 (5)
C2—C3—C4—C4A	−2.14 (8)	C4—C4A—C10A—N1	−0.56 (9)
C11—C3—C4—C4A	180.00 (5)	C5—C4A—C10A—N1	−178.82 (6)
N4—C4—C4A—C10A	−176.96 (5)	C4—C4A—C10A—O10	178.74 (5)
C3—C4—C4A—C10A	1.60 (8)	C5—C4A—C10A—O10	0.48 (9)
N4—C4—C4A—C5	1.29 (8)	C9A—O10—C10A—N1	168.16 (5)
C3—C4—C4A—C5	179.84 (5)	C9A—O10—C10A—C4A	−11.26 (9)
C10A—C4A—C5—C5A	11.45 (7)	C5A—C5—C21—C26	−52.13 (7)
C4—C4A—C5—C5A	−166.67 (5)	C4A—C5—C21—C26	69.23 (6)
C10A—C4A—C5—C21	−110.63 (6)	C5A—C5—C21—C22	128.86 (5)
C4—C4A—C5—C21	71.24 (6)	C4A—C5—C21—C22	−109.79 (6)
C4A—C5—C5A—C9A	−14.61 (7)	C26—C21—C22—C23	−1.52 (8)
C21—C5—C5A—C9A	108.55 (6)	C5—C21—C22—C23	177.51 (5)
C4A—C5—C5A—C6	164.97 (5)	C21—C22—C23—O2	−178.88 (5)
C21—C5—C5A—C6	−71.88 (6)	C21—C22—C23—C24	1.67 (9)
C9A—C5A—C6—O1	169.75 (6)	C22—C23—O2—C27	−3.02 (9)
C5—C5A—C6—O1	−9.86 (8)	C24—C23—O2—C27	176.45 (6)
C9A—C5A—C6—C7	−8.71 (8)	O2—C23—C24—O3	−1.81 (9)
C5—C5A—C6—C7	171.68 (5)	C22—C23—C24—O3	177.69 (6)
O1—C6—C7—C8	160.51 (6)	O2—C23—C24—C25	179.90 (6)
C5A—C6—C7—C8	−21.02 (8)	C22—C23—C24—C25	−0.60 (9)
C6—C7—C8—C13	169.85 (5)	O3—C24—C25—C26	−178.96 (6)
C6—C7—C8—C12	−70.19 (7)	C23—C24—C25—C26	−0.59 (9)
C6—C7—C8—C9	50.42 (7)	C22—C21—C26—C25	0.33 (9)
C13—C8—C9—C9A	−170.71 (6)	C5—C21—C26—C25	−178.70 (5)
C7—C8—C9—C9A	−51.84 (7)	C24—C25—C26—C21	0.73 (9)
C12—C8—C9—C9A	69.39 (7)	O98—C97—N99—C98	−0.18 (11)
C6—C5A—C9A—O10	−173.91 (5)	O98—C97—N99—C99	179.44 (7)
C5—C5A—C9A—O10	5.67 (9)		

### Hydrogen-bond geometry (Å, º)

**Table:**

*D*—H···*A*	*D*—H	H···*A*	*D*···*A*	*D*—H···*A*
N2—H01···O1^i^	0.886 (13)	2.268 (13)	3.1492 (7)	173.6 (11)
N2—H02···O98	0.885 (13)	2.170 (13)	2.9167 (8)	141.8 (11)
N4—H03···O98^ii^	0.836 (13)	2.590 (12)	3.2891 (8)	142.0 (11)
N4—H04···N3^iii^	0.863 (13)	2.237 (13)	3.0413 (8)	154.9 (12)
O3—H05···O99	0.879 (14)	1.816 (14)	2.6399 (7)	155.2 (12)
O99—H06···O1^iv^	0.856 (14)	1.944 (14)	2.7944 (7)	172.0 (13)
O99—H07···N1^v^	0.877 (14)	2.012 (14)	2.8699 (7)	165.5 (13)
O99—H07···O10^v^	0.877 (14)	2.519 (14)	3.2180 (7)	137.2 (11)
C22—H22···O98^ii^	0.95	2.39	3.3210 (8)	167

Symmetry codes: (i) *x*−1, *y*, *z*; (ii) *x*+1, *y*, *z*; (iii) −*x*+1, −*y*+2, −*z*+1; (iv) −*x*+5/2, *y*+1/2, −*z*+3/2; (v) −*x*+3/2, *y*+1/2, −*z*+3/2.

## References

[bb1] Ahmed, E. A., Elgemeie, G. H. & Ahmed, K. A. (2022). *Pigm. Resin Technol.* **51**, 1–5.

[bb2] Azzam, R. A., Elgemeie, G. H., Elsayed, R. E. & Jones, P. G. (2017). *Acta Cryst.* E**73**, 1820–1822. 10.1107/S2056989017015778 PMC5730231 29250394

[bb3] Bruker (1998). *XP*. Bruker Analytical X–Ray Instruments, Madison, Wisconsin, USA.

[bb4] Bruno, I. J., Cole, J. C., Edgington, P. R., Kessler, M., Macrae, C. F., McCabe, P., Pearson, J. & Taylor, R. (2002). *Acta Cryst.* B**58**, 389–397. 10.1107/s0108768102003324 12037360

[bb5] Elgemeie, G. E. H., Farag, D. S. & Jones, P. G. (1998*a*). *Acta Cryst.* C**54**, 1466–1468.

[bb6] Elgemeie, G. E. H., Fathy, N. M. & Jones, P. G. (1998*b*). *Acta Cryst.* C**54**, 1314–1316.

[bb7] Elgemeie, G. H., Mohamed, R. A., Hussein, H. A. & Jones, P. G. (2015). *Acta Cryst.* E**71**, 1322–1324. 10.1107/S2056989015018903 PMC4645039 26594501

[bb8] Fleming, F. F. & Wang, Q. Z. (2003). *Chem. Rev.* **103**, 2035–2078. 10.1021/cr020045d 12744700

[bb9] Groom, C. R., Bruno, I. J., Lightfoot, M. P. & Ward, S. C. (2016). *Acta Cryst.* B**72**, 171–179. 10.1107/S2052520616003954 PMC4822653 27048719

[bb10] Han, G.-F., Zhao, L.-J., Chen, L.-Z., Du, J.-W. & Wang, Z.-X. (2015). *J. Heterocycl. Chem.* **52**, 1219–1225.

[bb11] Hebishy, A. M. S., Elgemeie, G. H., Ali, R. A. E. & Jones, P. G. (2022). *Acta Cryst.* E**78**, 638–641. 10.1107/S2056989022005199 PMC9431781 36072138

[bb12] Hebishy, A. M. S., Elgemeie, G. H., Gouda, L. M. & Jones, P. G. (2023). *Acta Cryst.* E**79**, 335–340. 10.1107/S2056989023001883 PMC10088300 37057025

[bb13] Huszthy, P., Vermes, B., Báthori, N. & Czugler, M. (2003). *Tetrahedron*, **59**, 9371–9377.

[bb14] Karpinska, J., Erxleben, A. & McArdle, P. (2011). *Cryst. Growth Des.* **11**, 2829–2838.

[bb15] Metwally, N. H., Elgemeie, G. H. & Fahmy, F. G. (2023). *ACS Omega*, **8**, 36636–36654. 10.1021/acsomega.3c01987 PMC10568744 37841136

[bb16] Mohamed-Ezzat, R. A., Elgemeie, G. H. & Jones, P. G. (2021). *Acta Cryst.* E**77**, 547–550. 10.1107/S2056989021004126 PMC8100259 34026262

[bb17] Rigaku OD (2022). *CrysAlis PRO*. Rigaku Oxford Diffraction, Yarnton, England.

[bb18] Sheldrick, G. M. (2015*a*). *Acta Cryst.* C**71**, 3–8.

[bb19] Sheldrick, G. M. (2015*b*). *Acta Cryst.* A**71**, 3–8.

[bb20] Tu, X., Fan, W., Hao, W., Jiang, B. & Tu, S. (2014). *ACS Comb. Sci.* **16**, 647–651. 10.1021/co500100c 25229308

[bb21] Upadhyay, D. B., Vala, R. M., Patel, S. G., Patel, P. J., Chi, C. & Patel, C. (2023). *J. Mol. Struct.* **1273**, 134305.

[bb22] Wang, C., Li, Y., Gong, M., Wu, Q., Zhang, J., Kim, J. K., Huang, M. & Wu, Y. (2016). *Org. Lett.* **18**, 4151–4153. 10.1021/acs.orglett.6b01871 27512940

[bb23] Zhang, W., Yang, C., Zhang, Z., Li, X. & Cheng, J. (2019). *Org. Lett.* **21**, 4137–4142. 10.1021/acs.orglett.9b01325 31094530

[bb24] Zhang, G., Zhang, C., Tian, Y. & Chen, F. (2023). *Org. Lett.* **25**, 917–922. 10.1021/acs.orglett.2c04185 36730786

